# Does the Gut Microbiota Modulate Host Physiology through Polymicrobial Biofilms?

**DOI:** 10.1264/jsme2.ME20037

**Published:** 2020-07-04

**Authors:** Jiayue Yang, Yongshou Yang, Manami Ishii, Mayuko Nagata, Wanping Aw, Nozomu Obana, Masaru Tomita, Nobuhiko Nomura, Shinji Fukuda

**Affiliations:** 1 Institute for Advanced Biosciences, Keio University, 246–2 Mizukami, Kakuganji, Tsuruoka, Yamagata 997–0052, Japan; 2 Systems Biology Program, Graduate School of Media and Governance, Keio University, 5322 Endo, Fujisawa, Kanagawa 252–0882, Japan; 3 Faculty of Environment and Information Studies, Keio University, 5322 Endo, Fujisawa, Kanagawa 252–0882, Japan; 4 Transborder Medical Research Center, University of Tsukuba, 1–1–1 Tennodai, Tsukuba, Ibaraki 305–8575, Japan; 5 Microbiology Research Center for Sustainability, University of Tsukuba, 1–1–1 Tennodai, Tsukuba, Ibaraki 305–8572, Japan; 6 Faculty of Life and Environmental Sciences, University of Tsukuba, 1–1–1 Tennodai, Tsukuba, Ibaraki 305–8575, Japan; 7 Intestinal Microbiota Project, Kanagawa Institute of Industrial Science and Technology, 3–25–13 Tonomachi, Kawasaki-ku, Kawasaki, Kanagawa 210–0821, Japan; 8 Metabologenomics, Inc., 246–2 Mizukami, Kakuganji, Tsuruoka, Yamagata 997–0052, Japan

**Keywords:** gut microbiota, metabolite, gut microbiota-host interaction, biofilm

## Abstract

Microbes inhabit various environments, such as soil, water environments, plants, and animals. Humans harbor a complex commensal microbial community in the gastrointestinal tract, which is known as the gut microbiota. The gut microbiota participates not only in various metabolic processes in the human body, it also plays a critical role in host immune responses. Gut microbes that inhabit the intestinal epithelial surface form polymicrobial biofilms. In the last decade, it has been widely reported that gut microbial biofilms and gut microbiota-derived products, such as metabolites and bacterial membrane vesicles, not only directly affect the host intestinal environment, but also indirectly influence the health of the host. In this review, we discuss the most recent findings from human and animal studies on the interactions between the gut microbiota and hosts, and their associations with various disorders, including inflammatory diseases, atopic dermatitis, metabolic disorders, and psychiatric and neurological diseases. The integrated approach of metabologenomics together with biofilm imaging may provide valuable insights into the gut microbiota and suggest remedies that may lead to a healthier society.

Microbes inhabit numerous environments, including soil, water environments, plants, and animals. Humans host various complex commensal microbe communities in and on their bodies, such as the oral microbiota, skin microbiota, and gut microbiota (The Integrative HMP Research Network Consortium, 2019). Although there are up to 37 trillion cells in the human body, approximately 40 trillion gut microbes inhabit the gastrointestinal tract (Sender *et al.*, 2016). The host employs a number of mechanisms to maintain intestinal homeostasis and prevent anomalous immune responses directed against the microbiota. One of these protective mechanisms involves a mucus layer that covers the epithelial surface of the colon, which functions as a barrier and separates epithelial cells from gut microbes (Tropini *et al.*, 2017) (Fig. 1A). The mucus layer prevents pathogens from reaching and persisting on intestinal epithelial surfaces and, thus, it is an important component of host immunity. This layer is constantly renewed and acts as a trap for both commensal residents and pathogens, preventing their access to the epithelium (Johansson *et al.*, 2014; Desai *et al.*, 2016). The gut microbiota is present in different microhabitats and metabolic niches in the mucus layer secreted from the gut, the mucosa, and the surfaces of digestive residues in the gut lumen (Macfarlane and Dillon, 2007). There are several indications that gut microbes inhabit the colonic mucus as a polymicrobial biofilm, which is an extracellular matrix-enclosed aggregate form of microbes (Hooper and Gordon, 2001; Sonnenburg *et al.*, 2004; Flemming *et al.*, 2016). Bacteria in biofilms cooperate or compete with each other and form complex communities (Tytgat *et al.*, 2019). Healthy mucosal biofilms benefit the host because they are involved in the exchange of nutrients on the epithelial surface, increase colonization resistance, and protect the host from invasion by intestinal pathogens (Hooper and Gordon, 2001; Sonnenburg *et al.*, 2004). The disruption of healthy mucosal biofilms leads to the nearby or direct contact of pathogenic invasive biofilms with colonic epithelial cells, resulting in inflammation (Swidsinski *et al.*, 2005; Dejea *et al.*, 2014; Desai *et al.*, 2016) (Fig. 1B).

Gut microbes were initially discovered by Antonie van Leeuwenhoek (Dobell, 1932), and various species of gut microbes were subsequently identified (Table 1). In the past 15 years, the development of next-generation sequencing technology has markedly contributed to knowledge pertaining to the gut microbiome (Table 1). The gut microbiota in healthy adults consists of four main phyla: *Actinobacteria*, *Bacteroidetes*, *Firmicutes*, and *Proteobacteria* (Fukuda and Ohno, 2014). Gut microbiome profiles vary among individuals, and these profiles may be classified into three “enterotypes” (Arumugam *et al.*, 2011). However, recent studies have suggested that the human gut microbiome has gradients of dominant taxa rather than discrete enterotypes and its profile may be influenced by lifestyle (Knights *et al.*, 2014). In addition, the gut microbiota interacts with the host, cross-talks with the host immune system, and not only directly affects the host intestinal environment, it also indirectly affects the health of the host (Fukuda and Ohno, 2014; Tytgat *et al.*, 2019). Pathogen- and microbial-associated molecular patterns (PAMPs and MAMPs, respectively; *e.g.*, lipopolysaccharide [LPS], peptidoglycans, and flagellin from bacterial cells) and metabolites produced by the gut microbiota are the key players that shape our immune system during these interactions (Levy *et al.*, 2017) (Table 2).

## MAMP- and metabolite-mediated gut microbiota-host interactions

**Fig. 2.**
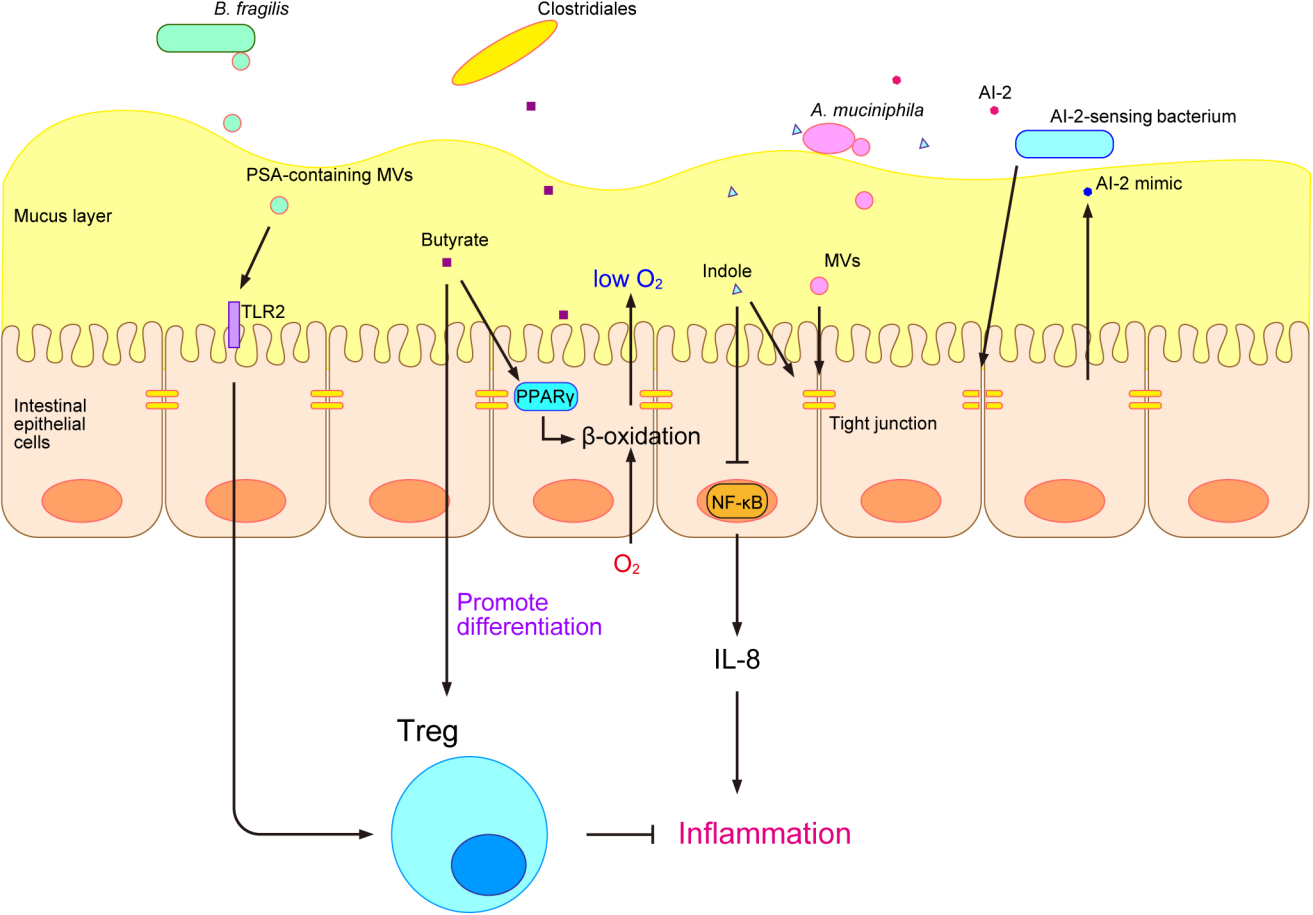
Schematic illustration depicting recently reported gut microbiota-host interactions in the intestinal epithelial surface. *Bacteroides fragilis* produce PSA and release it through OMVs, which promote Treg activity and anti-inflammatory cytokine production through TLR2 (Shen *et al.*, 2012). *Akkermansia muciniphila*-derived MVs ameliorate T2D in a mouse model by enhancing tight-junction function, thereby reducing gut permeability (Chelakkot *et al.*, 2018). Clostridiales produce butyrate and induce the differentiation of colonic Treg (Furusawa *et al.*, 2013). Butyrate also maintains the anaerobic environment in the colon by activating β-oxidation through PPARγ and consequently protecting the host from pathogenic proteobacteria (Byndloss *et al.*, 2017). The bacterial signaling molecule indole works as an interkingdom communication signal and increases tight-junction resistance while decreasing nuclear factor-κB (NF-κB) and the proinflammatory chemokine interleukin-8 (IL-8) in epithelial cells *in vitro* (Bansal *et al.*, 2010). Epithelial cells sense the disruption of tight junctions by bacteria and produce a molecule that mimics the bacterial signaling molecule AI-2, which may be detected by the bacterial AI-2 receptor and activates gene regulation controlled by AI-2 signaling (Ismail *et al.*, 2016).

Gut microbes interact with the host via gut microbe-derived products (Levy *et al.*, 2017). Gut microbe-derived MAMPs are recognized by pattern recognition receptors (PRRs), such as toll-like receptors (TLRs) and nucleotide-binding oligomerization domain-like receptors (NLRs), on epithelial cells (Cani, 2018). TLRs are transmembrane proteins that localize to the host cell surface, whereas NLRs are cytosolic proteins (Cani, 2018). A stimulation from gut commensal microbes shapes and trains the immune system. MAMPs are recognized by PRRs and induce adaptive responses by the immune system, resulting in the memory effect of innate immune cells and enhancements in the immune defenses of the host (Quintin *et al.*, 2012; Saeed *et al.*, 2014; Netea *et al.*, 2016). Although the immune stimulation by MAMPs has been extensively examined, this stimulation may also be caused by another type of microbe-derived substance called bacterial membrane vesicles (MVs). Bacterial MVs are “bubble”-like membrane structures released by bacteria that range between 20 and 400 nm in diameter (Toyofuku *et al.*, 2019). Many gut microbes and pathogens have been reported to produce MVs (Shen *et al.*, 2012; Obana *et al.*, 2017; Canas *et al.*, 2018; Chelakkot *et al.*, 2018). *Bacteroides fragilis* is a commensal microbe in the human gastrointestinal tract that produces a type of capsular exopolysaccharide called polysaccharide A (PSA), which has an immunomodulatory function. PSA was previously shown to be effective in animal models of inflammatory bowel disease (IBD) and multiple sclerosis (Shen *et al.*, 2012). PSA is carried by the MVs of *B. fragilis* and is delivered to the host immune system to promote regulatory T-cell (Treg) activity and anti-inflammatory cytokine production through TLR2 (Shen *et al.*, 2012) (Fig. 2). In addition, *Akkermansia muciniphila*-derived MVs are present at higher amounts in healthy individuals than in patients with type 2 diabetes (T2D). The administration of *A. muciniphila*-derived MVs ameliorated T2D in a mouse model by enhancing the tight-junction function of epithelial cells, which act as a barrier and reduce gut permeability (Chelakkot *et al.*, 2018) (Fig. 2).

The development of metabolome analysis technologies has allowed us to understand that host-gut microbe interactions occur not only via microbe-derived substances, but also through various microbial metabolites, such as trimethylamine N-oxide (TMAO), secondary bile acids, and short-chain fatty acids (SCFAs) (Fukuda and Ohno, 2014). TMAO is produced by gut microbes from dietary phosphatidylcholine and promotes atherosclerosis (Wang *et al.*, 2011). Bile acids are synthesized in the liver, secreted into the lumen of the small intestine, and facilitate the emulsification and absorption of lipids (Jia *et al.*, 2018). Gut microbes convert bile acids into various secondary bile acids, which have been reported to exert inflammatory and carcinogenic effects (Jia *et al.*, 2018). SCFAs, such as propionate, lactate, and butyrate, have been extensively examined and perform various functions in the host (Brown *et al.*, 2003; Furusawa *et al.*, 2013; Okada *et al.*, 2013; Smith *et al.*, 2013; Byndloss *et al.*, 2017). Propionate and other SCFAs activate the orphan G protein-coupled receptors GPR41 and GPR43 and affect host energy metabolism through intestinal peptides, such as glucagon-like peptide-1 (GLP-1) (Brown *et al.*, 2003). We previously showed that lactate induced the turnover of colonic epithelial cells in mice that were fed after starvation and promoted tumorigenesis in the colon (Okada *et al.*, 2013). Furthermore, butyrate was shown to have an immunomodulatory function in a mouse model and induced the differentiation of colonic Treg (Furusawa *et al.*, 2013) (Fig. 2). It also suppressed the growth of Enterobacteriaceae and protected the murine gut from *Salmonella* and *Escherichia coli* infections by activating β-oxidation and limiting luminal oxygen levels through the intracellular butyrate sensor peroxisome proliferator-activated receptor γ (PPARγ) (Byndloss *et al.*, 2017) (Fig. 2). To obtain a more detailed understanding of the relationship between the gut microbiome and gut metabolites, we developed an integrated omics analysis called metabologenomics, which is a method that includes both microbiome and metabolome analyses (Ishii *et al.*, 2018). This metabologenomic approach enables us to examine the complex network among the gut microbiota, gut microbiota-derived metabolites, and the host, thereby providing more comprehensive information about the gut environment (Ishii *et al.*, 2018). An analysis of the localization of various gut microbes in polymicrobial biofilms may provide novel insights into the metabolic network in the gut environment because previous studies suggested that gut microbes engage in cross-feeding in the gut environment (Belenguer *et al.*, 2006; Bunesova *et al.*, 2018; Pareek *et al.*, 2019), and biofilms are one of the locations at which cross-feeding occurs (Sztajer *et al.*, 2014; Sakanaka *et al.*, 2015).

In addition to microbial metabolites, bacterial cell-to-cell signaling molecules are also involved in interactions with the host. Bacteria produce several types of cell-to-cell signaling molecules for intraspecies and interspecies communication and regulate their gene expression and behavior (Yang *et al.*, 2017a; Mukherjee and Bassler, 2019). The tryptophan metabolite indole is a major signaling molecule that is produced by various gut microbes and is involved in bacterial interspecies communication as well as interkingdom communication between the host and gut microbes (Jaglin *et al.*, 2018). Indole increases the expression of genes involved in the function of the colonic mucosal barrier and in mucin secretion and increases tight-junction resistance while decreasing nuclear factor-κB (NF-κB) and the proinflammatory chemokine interleukin-8 (IL-8) in epithelial cells *in vitro* (Bansal *et al.*, 2010) (Fig. 2). Short-term exposure to indole promotes GLP-1 secretion by mouse colonic L cells through the inhibition of K^+^ channels, whereas long-term exposure decreases its secretion by blocking NADH dehydrogenase (Chimerel *et al.*, 2014). Autoinducer-2 (AI-2) is another cell-to-cell signaling molecule that modulates virulence production, biofilm formation, and gut colonization (Thompson *et al.*, 2015). Its production has been reported in many bacteria, including various gut microbes (Thompson *et al.*, 2015). AI-2 signaling has both interspecies and interkingdom communication functions. Notably, in the interspecies signaling of gut microbes, *Blautia obeum* has been suggested to protect the murine gut from *Vibrio cholerae* infection through AI-2 signaling (Hsiao *et al.*, 2014). AI-2 may also alter the composition of the gut microbiota in antibiotic-treated mice and increase the abundance of *Firmicutes*, one of the major phyla of gut microbes (Thompson *et al.*, 2015). In gut interkingdom signaling, epithelial cells sense the disruption of tight junctions by bacteria and produce a molecule that mimics AI-2, which may be detected by the bacterial AI-2 receptor and activates gene regulation controlled by AI-2 signaling (Ismail *et al.*, 2016) (Fig. 2). Previous studies revealed that AI-2 signaling modulated biofilm formation by gut microbes *in vitro* (González Barrios *et al.*, 2006; Ðapa *et al.*, 2013; Laganenka and Sourjik, 2018); therefore, the host appears to use AI-2 signaling to maintain the composition of its mucosal biofilms.

## Gut microbiota and host health

### Gut microbiota and gastrointestinal tract infection and inflammation

The gut microbiota maintains homeostasis in the gastrointestinal tract, and the disruption of this balance results in infection and inflammation (Levy *et al.*, 2017). We previously demonstrated that *Bifidobacterium longum* subsp. *longum* JCM 1217^T^ produced acetate and protected host mice from *E. coli* O157:H7 infection by up-regulating gut epithelial barrier function to block the translocation of the Shiga toxin from the gut lumen into the blood (Fukuda *et al.*, 2011). In addition, Clostridiales contributed to resistance to pathogen colonization and protected neonatal mice from infection (Kim *et al.*, 2017b). In gnotobiotic mice, the restriction of dietary fiber decreased the nutrient supply to the colon, promoted the growth of mucin-degrading *A. muciniphila*, and reduced the thickness of the colonic mucus barrier, which resulted in easy access and infection by the mucosal pathogen *Citrobacter rodentium* (Desai *et al.*, 2016).

IBD encompasses a group of autoimmune diseases in the gastrointestinal tract and mainly consists of Crohn’s disease and ulcerative colitis. The gut microbiota is related to IBD; the transition from symbiosis to dysbiosis in the gut results in gastric chronic inflammation (Nagao-Kitamoto and Kamada, 2017). Various species have been reported to cause inflammation. Although commensal *B. fragilis* is considered to be beneficial for IBD, enterotoxigenic strains of *B. fragilis* (ETBF) have been detected in the stools of 13.3% of IBD patients versus 2.9% of control groups (Nagao-Kitamoto and Kamada, 2017). ETBF produce toxins that bind to colonic epithelial cells, disrupt E-cadherin and the mucosal barrier, and increase IL-8 production, which results in inflammation (Nagao-Kitamoto and Kamada, 2017). Microscopic observations of gut samples from IBD patients revealed invasive biofilms abundant with *B. fragilis* covering the entire mucosa and in epithelial crypts (Swidsinski *et al.*, 2005). Furthermore, colonization of the colon by the oral pathogen *Klebsiella pneumoniae* resulted in IBD (Atarashi *et al.*, 2017). *K. pneumoniae* colonization results in the accumulation of T helper 1 (Th1) cells and a colitis phenotype in gnotobiotic mice. Furthermore, a metagenomic data analysis showed that *Klebsiella* was significantly more abundant in the feces of IBD patients, which provides further support for the involvement of this pathogen in the pathogenesis of IBD (Atarashi *et al.*, 2017).

Gut microbes and their polymicrobial invasive biofilms strongly correlated with the incidence of colorectal cancer (CRC) (Dejea *et al.*, 2014). *Fusobacterium*, a tumorigenic microbe, is enriched in CRC tumor tissue. The fluorescent *in situ* hybridization (FISH) staining of proximal CRC clinical samples showed that *Fusobacterium*-containing polymicrobial invasive biofilms existed directly on tumor tissue. Furthermore, clinical samples of tissues containing biofilms had lower levels of E-cadherin and higher expression levels of the inflammatory factor IL-6 and its activator Stat3 than those without biofilms (Dejea *et al.*, 2014). The same group also discovered that biofilm communities on the colon epithelium prepared from CRC patients and even healthy individuals were carcinogenic in murine CRC models (Tomkovich *et al.*, 2019). Moreover, biofilm-forming bacteria from CRC patients interacted with the host and altered host microRNA expression during the development of CRC in murine CRC models (Tomkovich *et al.*, 2020). One species of *Fusobacterium*, *Fusobacterium nucleatum*, is frequently detected in liver metastases of *Fusobacterium*-associated CRC (Bullman *et al.*, 2017). A treatment with the antibiotic metronidazole reduced the size of tumors in a mouse model of CRC, indicating that *F. nucleatum* is involved in CRC tumor growth (Bullman *et al.*, 2017). *F. nucleatum* may also induce TLR4 and MYD88 signaling and activate autophagy in CRC, resulting in chemoresistance (Yu *et al.*, 2017). We recently reported that *F. nucleatum* elevated the present form of carcinoma to a more advanced stage of CRC, while other bacteria in the early stage of CRC, namely, *Atopobium parvulum* and *Actinomyces odontolyticus*, co-occurred in intramucosal carcinomas (Yachida *et al.*, 2019). ETBF and polyketide synthase (*pks*) genotoxic island-containing (*pks*+) *E. coli* have also been identified as tumorigenic bacteria (Dejea *et al.*, 2018). The *pks* genotoxic island encodes the biosynthesis genes of a microbial carcinogen called colibactin, which alkylates DNA and forms colibactin-DNA adducts, resulting in DNA damage and genotoxicity (Wilson *et al.*, 2019). *pks*+ *E. coli* and ETBF co-colonize the colons of patients with familial adenomatous polyposis, which is caused by a hereditary mutation (Dejea *et al.*, 2018). A FISH analysis of clinical samples showed that *pks*+ *E. coli* and ETBF formed invasive biofilms on the mucosal tissues of FAP patients, and ETBF enhanced *pks*+ *E. coli* colonization in the colons of mice (Dejea *et al.*, 2018). Co-colonization in mice increased DNA damage in colonic epithelial cells, colonic tumor formation, and mortality. This tumorigenic effect was attributed to IL-17-induced inflammation (Dejea *et al.*, 2018). Taking these findings from CRC and IBD research into consideration, further studies on the comprehensive spatial information and microbiome compositions of polymicrobial biofilms are important for advancing the development of gut microbiota-targeted treatments.

### Gut microbiota and atopic dermatitis/immunology

Atopic dermatitis (AD) is a skin disease caused by chronic inflammation and is characterized by severe itching, redness, and eczematous skin lesions (Novak *et al.*, 2003). AD is initiated by the activation of T helper 2 (Th2) cells and suppression of Th1 (Grewe *et al.*, 1998). *Staphylococcus aureus* interacts with various cells of the cutaneous immune system to penetrate the epidermis and dermis and perpetuate chronic inflammation (Leyden *et al.*, 1974; Breuer *et al.*, 2002). Alterations in the gut microbiota influence the immune system balance via metabolite production, which may cause an inflamed microenvironment in the specific microbiome of the gut (Zeng *et al.*, 2017; Lee *et al.*, 2018). AD was previously shown to be induced when enteric bacteria from AD mice were transplanted into healthy sterile mice (Zachariassen *et al.*, 2017). *Faecalibacterium*, *Oscillospira*, *Bacteroides*, *Parabacteroides*, and *Sutterella* all have potential as gut microbe biomarkers for AD (Koga *et al.*, 2016; Reddel *et al.*, 2019). The abundance of *Clostridium difficile*, *E. coli*, and *S. aureus* was found to be higher in the gut microbiota of AD patients than in that of healthy individuals, whereas the amounts of *Bifidobacterium* and *Bacteroides* were lower (Kirjavainen *et al.*, 2002; Penders *et al.*, 2006; Abrahamsson *et al.*, 2012; Nylund *et al.*, 2015; Lee *et al.*, 2016). In addition, the SCFA-producing gut microbiota was confirmed to be present in a larger proportion of AD patients than healthy individuals (Song *et al.*, 2016).

In research on allergies in children who consume yogurt, yogurt consumption correlated with the prevention of AD (Shoda *et al.*, 2017). Moreover, *Faecalibacterium prausnitzii* attenuated the symptoms of AD (Song *et al.*, 2016). Similar findings were observed when *Lactobacillus sakei* WIKIM30 was orally administered to BALB/c mice with induced AD (Kwon *et al.*, 2018). WIKIM30 regulated Th2 and ameliorated AD by increasing the relative abundance of the gut microbiota responsible for the generation of Treg. *Lactococcus chungangensis* CAU 28, which is in cream cheese, was found to attenuate AD symptoms because it contributed to the suppression of Treg and Th2 immune responses by adjusting SCFAs and the gut microbiota and reducing the number of eosinophils and mast cells as well as immunoglobulin E levels (Kim *et al.*, 2019). In the feces of 11 healthy children and 28 infants who developed AD, the severity of AD inversely correlated with gut microbiota diversity and butyric acid-producing bacteria (Nylund *et al.*, 2015).

A recent study suggested a relationship between AD and T helper 17 (Th17) cells (Sugaya, 2020). IL-17 and IL-22 are cytokines that are secreted by Th17 and serve as its marker (Ivanov *et al.*, 2009). A previous study reported that the number of IL-22-producing Th17 cells was significantly increased in the skin of AD patients (Nograles *et al.*, 2009). In another study, the number of IL-17-producing T cells was found to be increased in the peripheral blood and acute lesional skin of AD patients (Koga *et al.*, 2008). In addition, Treg were shown to attenuate inflammation in AD mice (Kalekar and Rosenblum, 2019). Although there is currently no information on gut microbial biofilms in the field of AD, the adherence of bacteria and mucosal biofilms may be important for the immunology of AD. For example, segmented filamentous bacterium (SFB) and enterohemorrhagic *E. coli* (EHEC), which induce the differentiation of Th17, and Clostridia, which induce the differentiation of Treg, are intestinal epithelium-associated or -adhering bacteria (Ivanov *et al.*, 2009; Atarashi *et al.*, 2013; Furusawa *et al.*, 2013; Atarashi *et al.*, 2015). Therefore, researchers need to focus more on the connections between mucosal biofilms and immunology.

### Gut microbiota and metabolic disorders

The prevalence of overweight and obese individuals is a growing epidemic health concern and affected an estimated 1.3 billion individuals worldwide in 2016 (NCD Risk Factor Collaboration, 2017). The progression of certain metabolic disorders, including T2D and atherosclerotic cardiovascular disease (ACVD), is linked to being overweight or obese. Obesity and T2D are both associated with a gut microbiota with an altered composition and function (Qin *et al.*, 2012; Harsch and Konturek, 2018). Although many studies have attempted to elucidate the roles of the gut microbiota in obesity, the precise mechanisms remain unclear because of the complexity of the relationship between the host and microbiota. Diet is one of the environmental factors contributing to obesity, and has been extensively examined in connection with the gut microbiota (Carmody *et al.*, 2015). For example, a Western diet, characterized by high fat and low dietary fiber, has been shown to influence the composition of the gut microbiota and reduce its diversity (He *et al.*, 2018). The findings of a recent study on pre-obese children suggested that the composition of the gut microbiota in conjunction with long-term dietary habits may be helpful for predicting the development of obesity in children (Rampelli *et al.*, 2018). These findings also indicated that an impaired gut microbiota causes metabolic dysfunction and ultimately obesity in the host (Rampelli *et al.*, 2018). The composition of the gut microbiota of obese animals and humans differs from that of lean subjects (Hartstra *et al.*, 2015; Castaner *et al.*, 2018). The ratio of the phyla *Firmicutes* and *Bacteroidetes* was increased in obese subjects and considered to be associated with higher energy absorption from food and elevated low-grade inflammation (Turnbaugh *et al.*, 2006). Additionally, metabolites of the gut microbiota, including SCFAs, LPS, and secondary bile acids, play a critical role in the modulation of metabolism and obesity (Baothman *et al.*, 2016). Increased levels of Enterobacteriaceae and *Streptococcus* spp. have been identified in the stools of ACVD patients by a metagenome-based study (Jie *et al.*, 2017). Furthermore, the gut bacterial metabolite TMAO produced from choline, l-carnitine, and phosphatidylcholine promoted the progression of ACVD (Wang *et al.*, 2011; Koeth *et al.*, 2013; Tang *et al.*, 2013).

By using culture-based approaches developed in the past decade to examine the gut microbiota (Sommer, 2015), it is possible to investigate the relationship between specific bacterial species and various conditions, including obesity. Endotoxin-producing *Enterobacter cloacae* has been shown to promote obesity and insulin resistance in germ-free mice (Fei and Zhao, 2013). Similarly, *Clostridium ramosum*, promoted diet-induced obesity, possibly by enhancing nutrient absorption (Woting *et al.*, 2014). In contrast, some bacterial species exert anti-obesogenic effects (Table 3). For example, supplementation with *A. muciniphila* ameliorated the symptoms of metabolic syndrome induced by a high-fat diet, including fat mass gain, chronic tissue inflammation, metabolic endotoxemia, and insulin resistance, and improved the inflammatory state and gut barrier (Everard *et al.*, 2013). Due to its critical role in the maintenance of metabolic homeostasis, *A. muciniphila* has been proposed as a next-generation probiotic to combat obesity, diabetes, and cardiometabolic disorders (Cani and de Vos, 2017). *Prevotella copri* performs carbohydrate fermentation from complex polysaccharides in the diet and contributes to better glucose tolerance; therefore, it is regarded as a beneficial bacterium in high-fiber dietary interventions (Kovatcheva-Datchary *et al.*, 2015; De Vadder *et al.*, 2016). Two species of the genus *Parabacteroides*, *Parabacteroides goldsteinii* and *Parabacteroides distasonis*, were found to have the potential to correct obesity-associated abnormalities in mice fed a high-fat diet (Wang *et al.*, 2019a; Wu *et al.*, 2019). These findings on the roles of specific bacterial species in the gut provide important insights for understanding intricate gut microbiota-host interactions. The identification of these pro- and anti-obesogenic bacteria and a more detailed understanding of the mechanisms underlying this disease will enable us to explore effective approaches for the treatment of metabolic disorders, including obesity. The link between gut microbial biofilms and metabolic disorders currently remains unclear. However, gut microbial biofilms may play a key role because their composition and structure were previously shown to be changed by different diets (Earle *et al.*, 2015).

### Gut microbiota and the gut-brain axis

The gut-brain axis is a system through which the gut microbiota affects brain development, neural activities, and brain disease (Mayer *et al.*, 2014). In recent years, the effects of this relatively unknown system on physical and neurological conditions have been attracting increasing attention.

Many diseases and phenomena have been associated with the gut-brain axis (Schroeder and Bäckhed, 2016; Ye *et al.*, 2018). One example is anxiety-like behavior. Recent studies reported that antibiotics alter the composition of the gut microbiota, which subsequently results in long-term changes in behavior (Burokas *et al.*, 2017; De Palma *et al.*, 2017). Another study showed the attenuation of anxiety-like behavior in the male offspring of pregnant mice exposed to a low dose of penicillin (Leclercq *et al.*, 2017). During defeat sessions in which control group mice and penicillin-treated mice were physically subjected to stress by unfamiliar male aggressors, the penicillin-treated group fought back and had fewer scars than control mice, indicating that a deficiency in gut microbes was associated with a reduced fear response (Leclercq *et al.*, 2017). Similarly, an imbalance in the hypothalamic-pituitary-adrenal axis caused by gut microbiome alterations may affect the neuroendocrine system, provoking anxiety-like behavior. After the application of a chronic restraint stress to germ-free and specific pathogen-free mice, specific pathogen-free mice exhibited more anxiety-like behavior than germ-free mice (Huo *et al.*, 2017). In addition, microbiota-derived metabolites were found to contribute to the mental health of the host and relieved psychosocial stress (van de Wouw *et al.*, 2018).

Autism spectrum disorder (ASD) is a severe neurodevelopmental disorder involving impaired social communication and interactions as well as repetitive behavior patterns. The gut microbiota has been suggested to play a critical role in the pathogenesis of ASD. The gut microbiota profiles of ASD patients markedly differ from those of typically developing controls (Kang *et al.*, 2013; Tomova *et al.*, 2015; Liu *et al.*, 2019). *Bifidobacterium*, *Blautia*, *Dialister*, *Prevotella*, *Veillonella*, and *Turicibacter* levels were consistently lower in ASD patients than in healthy controls, whereas *Lactobacillus*, *Bacteroides*, *Desulfovibrio*, and *Clostridium* levels were higher (Yang *et al.*, 2018; Liu *et al.*, 2019). Similarly, an altered gut microbiota composition was observed in ASD mice (Hsiao *et al.*, 2013; Buffington *et al.*, 2016; Kim *et al.*, 2017a). An altered gut microbiota composition resulted in the production of different metabolites, which may influence the progression of ASD (Wang *et al.*, 2019b). Children with ASD had significantly higher levels of *Actinobacteria*, but significantly lower species richness than those exhibiting typical development (Wang *et al.*, 2019b). Supplementation with probiotics and fecal microbiota transplantation alleviated the symptoms of ASD in children (Steenbergen *et al.*, 2015; Kang *et al.*, 2017). Interestingly, specific bacterial species, such as *B. fragilis* and *Lactobacillus reuteri*, have therapeutic potential in animal models of ASD (Hsiao *et al.*, 2013; Buffington *et al.*, 2016).

The effects of gut microbial communities on physical and neurological conditions have also been examined in Alzheimer’s disease (AZD). Accounting for ~70% of all dementia cases, AZD is the most common form of dementia (Ko *et al.*, 2019). The diversity of the gut microbiota was previously reported to be lower in AZD patients than in age- and sex-matched control individuals (Vogt *et al.*, 2017). Bile acids also play an important role in the development of AZD. A recent study compared the bile acid profiles of healthy adults, adults with early mild cognitive impairment, adults with late mild cognitive impairment, and AZD patients, and found that primary bile acid levels were lower and microbiota-produced secondary bile acid levels were higher in subjects with cognitive impairment (MahmoudianDehkordi *et al.*, 2019). Therefore, specific changes in the gut microbial community were suggested to alter the levels of secondary bile acids, conceivably contributing to the pathogenesis of AZD (MahmoudianDehkordi *et al.*, 2019).

Similar gut microbiota alterations have been observed in Parkinson’s disease (PD) patients (Scheperjans *et al.*, 2015; Hill-Burns *et al.*, 2017). The symptoms of PD, a progressive nervous system disorder, include motor deficits, such as tremors, rigid muscles, and bradykinesia (Nalls *et al.*, 2014). In PD patients, gut bacteria from the family Prevotellaceae were 77.6% lower than in healthy individuals (Scheperjans *et al.*, 2015). Another study transplanted the gut microbiota from PD patients to germ-free mice, and detected physical impairments due to neurodegeneration in recipient mice (Sampson *et al.*, 2016). Collectively, these findings indicate that gut microbes contribute to the progression of PD.

Gut-innervating nociceptor neurons have been shown to inhibit *Salmonella* infection through the modulation of Peyer’s Patch microfold cells and gut adherence bacterium SFB levels (Lai *et al.*, 2020). Although the relationships between bacterial adherence or biofilm formation and the brain currently remain unclear, some relationships appear to exist between the adherence of gut microbes and the neurological system.

### Conclusions

**Fig. 3.**
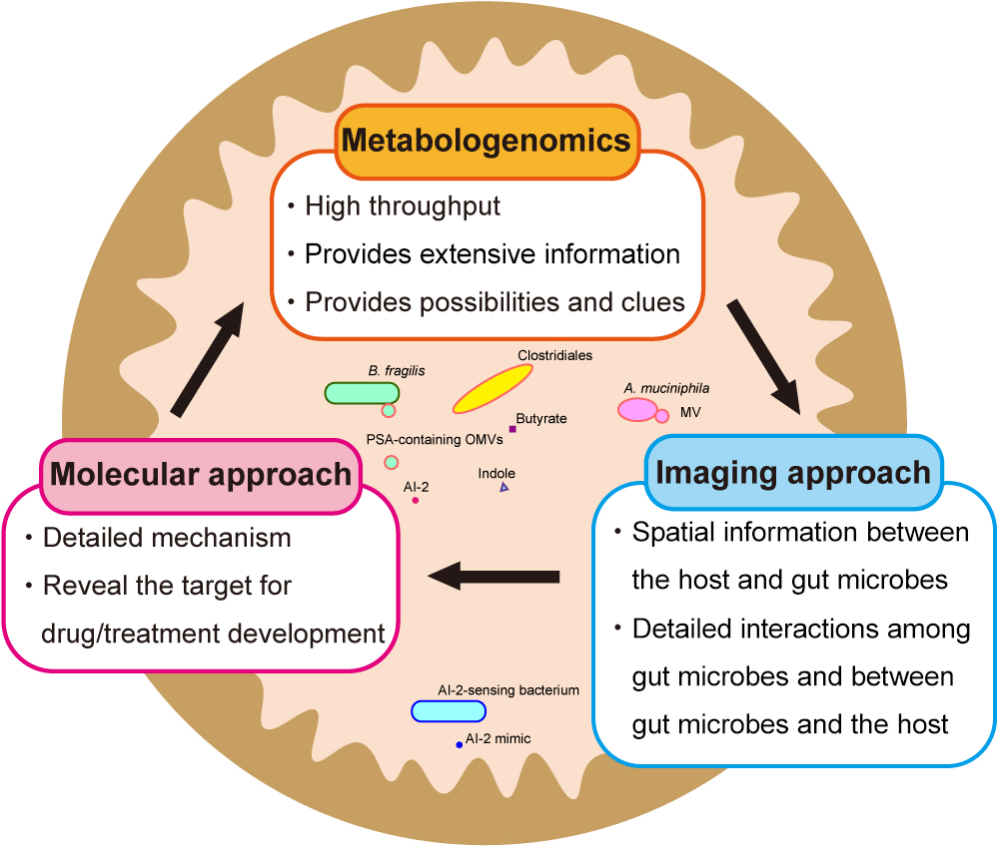
A synergistic approach integrating metabologenomics, imaging, and molecular technologies. The integrated omics analysis method, metabologenomics provides us with extensive information on the relationships between microbiota, metabolites, and the host with many possibilities and clues for further research (Ishii *et al.*, 2018). The imaging approach enables us to gain spatial information between the host and gut microbes, and a more detailed understanding of the interactions among gut microbes as well as gut microbes with the host. The molecular approach reveals detailed mechanisms and targets for the drug/treatment development. Novel synergy between metabologenomics, imaging approaches, and molecular approaches will allow us to elucidate the mechanisms underlying gut microbe-host interactions and host immune defenses on the intestinal mucosal barrier in order to develop better solutions for human health issues.

Various omics technologies enable us to obtain extensive information in a high-throughput manner in gut microbe research. The development of the integrated omics analysis method, metabologenomics, has facilitated research on complex gut microbiota communities and gut microbiota-host interaction networks (Ishii *et al.*, 2018). Large cohort studies and high-throughput omics techniques may be commonly used in this area of study. However, difficulties may be associated with investigating host-microbiota interactions by solely relying on an omics analysis. As shown in studies on IBD and CRC, spatial information on the intestinal mucosal surface is important for examining host-microbiota interactions. Therefore, gut microbiota research may need to shift its focus from the compositional traits and variations of gut microbiota and metabolites to integrating them into the spatial and temporal dynamics of gut microbial biofilms. Therefore, it is important to incorporate spatio-temporal characterizations using microscopic imaging and molecular biology analyses because they may contribute to clarifying the detailed mechanisms underlying gut microbiota-host interactions and host immune defenses on the intestinal mucosal barrier and also reveal new targets for drug/treatment development (Fig. 3) (Swidsinski *et al.*, 2005; Dejea *et al.*, 2014; Atarashi *et al.*, 2017; Bullman *et al.*, 2017; Yu *et al.*, 2017; Dejea *et al.*, 2018; Wilson *et al.*, 2019). Novel synergy among these approaches will revolutionize the field of gut microbiology, thereby allowing us to make more contributions toward maintaining and promoting optimal host health for a higher quality of life.

## Citation

Yang, J., Yang, Y., Ishii, M., Nagata, M., Aw, W., Obana, N., et al. (2020) Does the Gut Microbiota Modulate Host Physiology through Polymicrobial Biofilms?. *Microbes Environ ***35**: ME20037.

https://doi.org/10.1264/jsme2.ME20037

## Figures and Tables

**Fig. 1.**
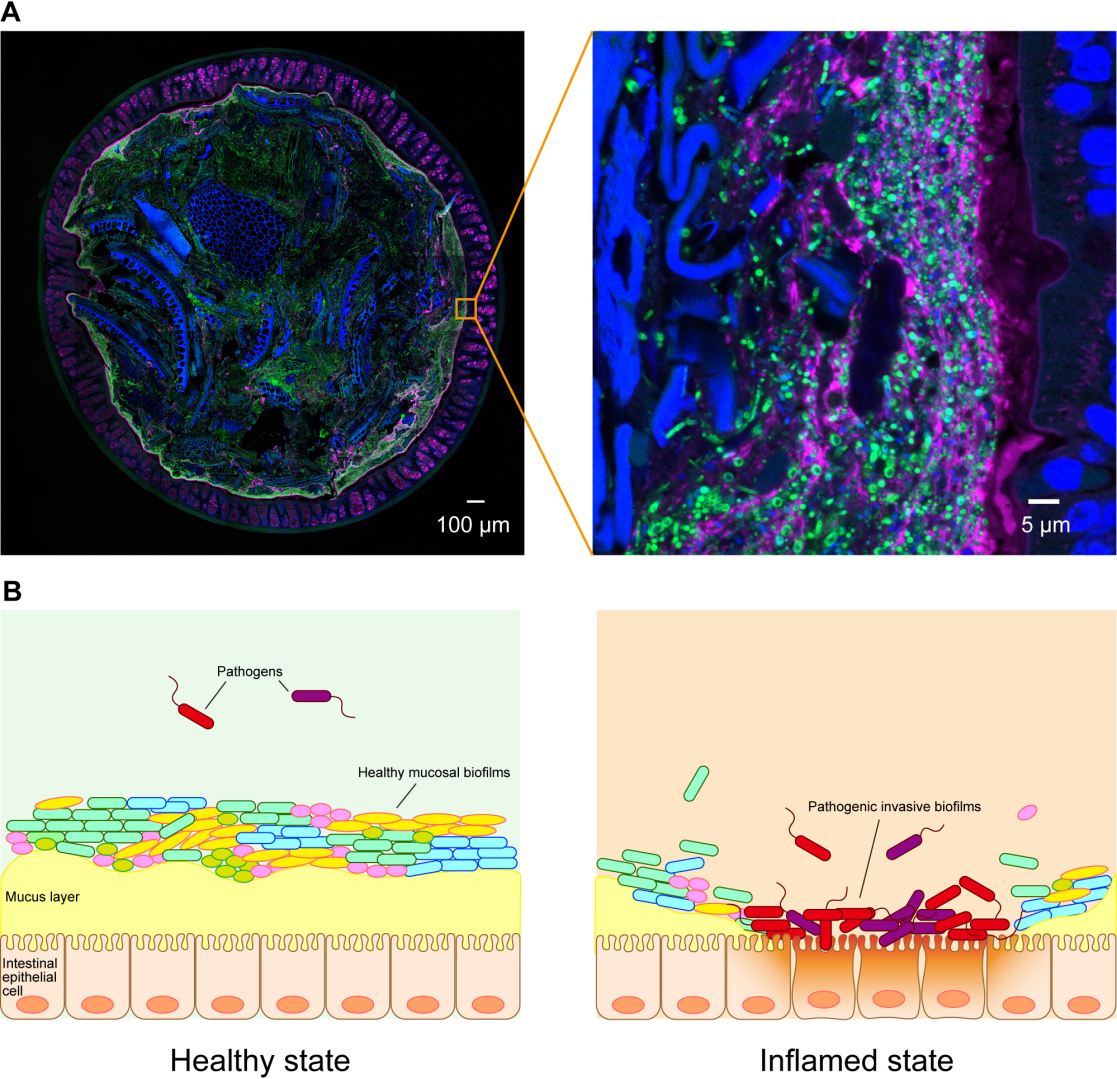
The interaction of gut microbial biofilms with the host. (A) Confocal microscopy fluorescent *in-situ* hybridization (FISH) images of the whole distal colon (left) and enlarged images of the epithelial surface (right). Blue: DNA stained with DAPI and the autofluorescence of dietary fibers; green: bacteria stained with the FAM-labeled Eub338 FISH probe; magenta: mucus stained with Alexa555-conjugated wheat germ agglutinin lectin. (B) Schematic images of healthy mucosal biofilms and the inflamed condition. In the healthy state, gut microbes inhabit colonic mucus as a polymicrobial biofilm (Hooper and Gordon, 2001; Sonnenburg *et al.*, 2004). In the inflamed state, colonic pathogens (red, purple) form pathogenic invasive biofilms in direct contact with colonic epithelial cells (Swidsinski *et al.*, 2005; Dejea *et al.*, 2014; Tytgat *et al.*, 2019). The left figure of part A was adapted with permission from Yodosha (Fukuda, 2019). The right figure of part A is the original photograph from our laboratory.

**Table 1.** Chronology of milestones in gut microbiota research

Year	Event	Reference
1670s~1680s	Discovery of gut microbes by Antonie van Leeuwenhoek	Dobell, 1932
1849	Discovery of segmented filamentous bacteria (SFB)	Leidy, 1849
1885	Discovery of *Escherichia coli*	Escherich, 1885
1900	Discovery of *Bifidobacterium bifidum*	Tissier, 1900
1900	Discovery of* Lactobacillus acidophilus*	Moro, 1900
1953	Discovery of the DNA double helix structure	Watson & Crick, 1953
1969	Establishment of the culture method of intestinal anaerobes	Mitsuoka *et al.*, 1969
1975	Invention of the sequencing method	Sanger & Coulson, 1975
1987	Invention of the PCR method	Mullis & Faloona, 1987
1990~2003	Human genome project	International Human Genome Sequencing Consortium, 2004
1991	Proposal of the classification of bacteria by 16S rRNA sequences	Weisburg *et al.*, 1991
2004	Sargasso Sea environmental genome sequencing project	Venter *et al.*, 2004
2005	Launched the first next-generation sequencer GS20 (454 Life Sciences)	Voelkerding *et al.*, 2009
2006	Study on the relationship between obesity and the gut microbiome	Turnbaugh *et al.*, 2006
2007	Metagenomic sequencing of the Japanese gut microbiome	Kurokawa *et al.*, 2007
2007	Metabolomic study on metabolites of *E. coli*	Ishii *et al.*, 2007
2008~2014	Human microbiome project	Human Microbiome Project Consortium (2012)
2009	Discovering the induction of Th17 differentiation by SFB	Ivanov *et al.*, 2009
2010	Proposing the concept of “Enterotypes”	Arumugam *et al.*, 2011
2011	Discovering the anti-infection ability of *Bifidobacterium* upon the *E. coli* O157 strain through acetate	Fukuda *et al.*, 2011
2013	Discovering that SCFAs regulate colonic Treg cell homeostasis	Smith *et al.*, 2013
2013	Discovering the colonic Treg differentiation induction ability of Clostridia-derived SCFA butyrate	Furusawa *et al.*, 2013
2013	Proposal of fecal microbiota transplantation treatment for *Clostridium difficile* infection	van Nood *et al.*, 2013
2014~2019	Integrative human microbiome project	iHMP Research Network Consortium, 2019

**Table 2.** Well-used acronyms in this study

Abbreviation	Full name
ACVD	atherosclerotic cardiovascular disease
AD	Atopic dermatitis
ASD	Autism spectrum disorder
AZD	Alzheimer’s disease
CRC	colorectal cancer
ETBF	enterotoxigenic strains of *B. fragilis*
FAP	familial adenomatous polyposis
GLP-1	glucagon-like peptide-1
IBD	inflammatory bowel disease
MAMPs	microbial-associated molecular patterns
MVs	membrane vesicles
NF-κB	nuclear factor-κB
PAMPs	pathogen-associated molecular patterns
PD	Parkinson’s disease
PRRs	pattern recognition receptors
PSA	polysaccharide A
SCFAs	short-chain fatty acids
SFB	segmented filamentous bacteria
T2D	type 2 diabetes
TLRs	Toll-like receptors
TMAO	trimethylamine N-oxide

**Table 3.** Roles of specific bacterial species with anti-obesogenic effects

Bacterium	Subject	Treatment period	Outcomes	Reference
*Akkermansia muciniphila*	Mice	4‍ ‍weeks	Alleviates high-fat diet-induced metabolic symptoms, including endotoxemia, fat mass gain, adipose tissue inflammation, and insulin resistance; improves inflammation, gut barrier function, and gut peptide secretion	Everard *et al.*, 2013
*Prevotella copri*	Mice	2‍ ‍weeks	Performs carbohydrate fermentation from complex polysaccharides in the diet and contributes to better glucose tolerance	Kovatcheva-Datchary *et al.*, 2015
*Bifidobacterium breve* B-3	Mice	8‍ ‍weeks	Reduces the accumulation of body weight and epididymal fat and alleviates serum levels of fasting glucose, cholesterol, and insulin	Kondo *et al.*, 2010
*Bifidobacterium breve* B-3	Pre-obesity adults	12‍ ‍weeks	Reduces fat mass and alleviates parameters associated with liver functions and inflammation, including γ-glutamyltranspeptidase and high-sensitivity C-reactive protein	Minami *et al.*, 2015
*Bifidobacterium pseudocatenulatum*	Mice	8‍ ‍weeks	Reduces body weight and fat mass, fasting glucose, and insulin resistance	Zhao *et al.*, 2018
*Lactobacillus gasseri*	Healthy adults with large visceral fat areas	12‍ ‍weeks	Reduces abdominal visceral fat areas, the body mass index, hip and waist circumferences, and body fat	Kadooka *et al.*, 2013
*Bacteroides uniformis*	Mice	7‍ ‍weeks	Suppresses body weight gain; improves liver function by reducing liver steatosis as well as liver cholesterol and triglycerides; reduces dietary fat absorption and reverses immune dysfunction	Gauffin Cano *et al.*, 2012
*Bacteroides acidifaciens*	Mice	10‍ ‍weeks	Suppresses body weight and fat mass; ameliorates insulin resistance; increases serum GLP-1 and decreases gut dipeptidyl peptidase-4	Yang *et al.*, 2017b
*Bacteroides thetaiotaomicron*	Mice	7‍ ‍weeks	In chow diet-fed mice, reduces fat mass and increases lean body mass; in high-fat diet-fed mice, suppresses body weight gain and adiposity, increases serum adiponectin and decreases leptin, up-regulates the expression of genes for fatty acid oxidation and lipolysis, and improves the inflammatory status	Liu *et al.*, 2017
*Eubacterium hallii*	*db/db* mice	5‍ ‍weeks	Decreases insulin sensitivity and increases energy expenditure and fecal butyrate concentrations	Udayappan *et al.*, 2016
*Parabacteroides goldsteinii*	Mice	8‍ ‍weeks	Ameliorates obesity and increases adipose tissue thermogenesis; enhances gut integrity; lowers the inflammatory status; increases insulin sensitivity	Wu *et al.*, 2019
*Parabacteroides distasonis*	Mice	5‍ ‍weeks	Reduces weight gain, hyperglycemia, and hepatic steatosis; alters the bile acid profile; increases gut gluconeogenesis and insulin sensitivity	Wang *et al.*, 2019a
